# Supplementary material for: Hybrid Models and Biological Model Reduction with PyDSTool
Source: PLoS Comput Biol. 2012 Aug 9;8(8):e1002628. doi: 10.1371/journal.pcbi.1002628 (PMC3415397; doi:10.1371/journal.pcbi.1002628)
Supplement: Text S4 — Complete source code for the PyDSTool package (version 0.88.120504). Includes API documentation and help files linking to web pages. This file is identical to the current public release on Sourceforge.net. (ZIP) [file pcbi.1002628.s004.zip › PyDSTool/html/PyDSTool.Toolbox.mechmatlib-module.html]

xml version="1.0" encoding="ascii"?


PyDSTool.Toolbox.mechmatlib


| Home | Trees | Indices | Help | PyDSTool |
| --- | --- | --- | --- | --- |

Package PyDSTool ::
Package Toolbox ::
Module mechmatlib

[hide private]

[frames] | no frames]

# Module mechmatlib

source code


|  |  |
| --- | --- |
| Functions | [hide private] |

|  |  |
| --- | --- |
| dot(a, b)  Dot product of two arrays. |  |

|  |  |
| --- | --- |
| augment\_3\_vector(v=array([ 0., 0., 0.]), free=False) | source code |

|  |  |
| --- | --- |
| augment\_3x3\_matrix(M=matrix([[ 1., 0., 0`...`, disp=array([ 0., 0., 0.]), free=False) | source code |

|  |  |
| --- | --- |
| make\_disp(r)  Make 3D displacement | source code |

|  |  |
| --- | --- |
| make\_rot(a)  Make 3D rotation (around z, then new x, then new y) | source code |

|  |  |
| --- | --- |
| make\_T(r, a)  Make general 3D transformation (displacement then rotation) | source code |

|  |  |
| --- | --- |
| cross(a, b)  Cross product of two 3D vectors | source code |


|  |  |
| --- | --- |
| Variables | [hide private] |

ZeroPos = `array([ 0., 0., 0., 1.])`

Id = `matrix([[ 1., 0., 0., 0...`


|  |  |
| --- | --- |
| Function Details | [hide private] |

|  |  |
| --- | --- |
| dot(a, b) |  |

```
Dot product of two arrays.

For 2-D arrays it is equivalent to matrix multiplication, and for 1-D
arrays to inner product of vectors (without complex conjugation). For
N dimensions it is a sum product over the last axis of `a` and
the second-to-last of `b`::

    dot(a, b)[i,j,k,m] = sum(a[i,j,:] * b[k,:,m])

Parameters
----------
a : array_like
    First argument.
b : array_like
    Second argument.

Returns
-------
output : ndarray
    Returns the dot product of `a` and `b`.  If `a` and `b` are both
    scalars or both 1-D arrays then a scalar is returned; otherwise
    an array is returned.

Raises
------
ValueError
    If the last dimension of `a` is not the same size as
    the second-to-last dimension of `b`.

See Also
--------
vdot : Complex-conjugating dot product.
tensordot : Sum products over arbitrary axes.

Examples
--------
>>> np.dot(3, 4)
12

Neither argument is complex-conjugated:

>>> np.dot([2j, 3j], [2j, 3j])
(-13+0j)

For 2-D arrays it's the matrix product:

>>> a = [[1, 0], [0, 1]]
>>> b = [[4, 1], [2, 2]]
>>> np.dot(a, b)
array([[4, 1],
       [2, 2]])

>>> a = np.arange(3*4*5*6).reshape((3,4,5,6))
>>> b = np.arange(3*4*5*6)[::-1].reshape((5,4,6,3))
>>> np.dot(a, b)[2,3,2,1,2,2]
499128
>>> sum(a[2,3,2,:] * b[1,2,:,2])
499128
```

  


|  |  |
| --- | --- |
| Variables Details | [hide private] |

### Id

Value:
:   ```
    matrix([[ 1.,  0.,  0.,  0.],
            [ 0.,  1.,  0.,  0.],
            [ 0.,  0.,  1.,  0.],
            [ 0.,  0.,  0.,  1.]], dtype=float32)
    ```

  


| Home | Trees | Indices | Help | PyDSTool |
| --- | --- | --- | --- | --- |

|  |  |
| --- | --- |
| Generated by Epydoc 3.0.1 on Fri May 4 15:24:04 2012 | http://epydoc.sourceforge.net |
